# Presynaptic External Calcium Signaling Involves the Calcium-Sensing Receptor in Neocortical Nerve Terminals

**DOI:** 10.1371/journal.pone.0008563

**Published:** 2010-01-05

**Authors:** Wenyan Chen, Jeremy B. Bergsman, Xiaohua Wang, Gawain Gilkey, Carol-Renée Pierpoint, Erin A. Daniel, Emmanuel M. Awumey, Philippe Dauban, Robert H. Dodd, Martial Ruat, Stephen M. Smith

**Affiliations:** 1 Division of Pulmonary & Critical Care Medicine, Oregon Health & Science University, Portland, Oregon, United States of America; 2 Julius L. Chambers Biomedical/Biotechnology Research Institute, North Carolina Central University, Durham, North Carolina, United States of America; 3 Centre National de la Recherche Scientifique, Institut de Chimie des Substances Naturelles, Gif-sur-Yvette, France; 4 Centre National de la Recherche Scientifique, Institut de Neurobiologie Alfred Fessard, Gif-sur-Yvette, France; INSERM U862, France

## Abstract

**Background:**

Nerve terminal invasion by an axonal spike activates voltage-gated channels, triggering calcium entry, vesicle fusion, and release of neurotransmitter. Ion channels activated at the terminal shape the presynaptic spike and so regulate the magnitude and duration of calcium entry. Consequently characterization of the functional properties of ion channels at nerve terminals is crucial to understand the regulation of transmitter release. Direct recordings from small neocortical nerve terminals have revealed that external [Ca^2+^] ([Ca^2+^]_o_) indirectly regulates a non-selective cation channel (NSCC) in neocortical nerve terminals via an unknown [Ca^2+^]_o_ sensor. Here, we identify the first component in a presynaptic calcium signaling pathway.

**Methodology/Principal Findings:**

By combining genetic and pharmacological approaches with direct patch-clamp recordings from small acutely isolated neocortical nerve terminals we identify the extracellular calcium sensor. Our results show that the calcium-sensing receptor (CaSR), a previously identified G-protein coupled receptor that is the mainstay in serum calcium homeostasis, is the extracellular calcium sensor in these acutely dissociated nerve terminals. The NSCC currents from reduced function mutant CaSR mice were less sensitive to changes in [Ca^2+^]_o_ than wild-type. Calindol, an allosteric CaSR agonist, reduced NSCC currents in direct terminal recordings in a dose-dependent and reversible manner. In contrast, glutamate and GABA did not affect the NSCC currents.

**Conclusions/Significance:**

Our experiments identify CaSR as the first component in the [Ca^2+^]_o_ sensor-NSCC signaling pathway in neocortical terminals. Decreases in [Ca^2+^]_o_ will depress synaptic transmission because of the exquisite sensitivity of transmitter release to [Ca^2+^]_o_ following its entry via voltage-activated Ca^2+^ channels. CaSR may detects such falls in [Ca^2+^]_o_ and increase action potential duration by increasing NSCC activity, thereby attenuating the impact of decreases in [Ca^2+^]_o_ on release probability. CaSR is positioned to detect the dynamic changes of [Ca^2+^]_o_ and provide presynaptic feedback that will alter brain excitability.

## Introduction

Neurotransmitter release from nerve terminals underlies synaptic communication in the brain. Invasion of the nerve terminal by an axonal spike activates voltage-gated channels, triggering calcium entry and exocytosis of transmitter-containing vesicles [1]. Release probability at a given synapse is dynamic; the ion channels activated at the terminal shape the presynaptic spike and so regulate the magnitude and duration of calcium entry [2], [3], [4]. Characterization of the functional properties of ion channels at nerve terminals is thus crucial to understand presynaptic regulation of transmitter release. Extension of patch clamp techniques to small, relatively inaccessible nerve terminals has substantially increased our understanding of presynaptic function at these important sites [4], [5]. One unexpected finding is that external [Ca^2+^] ([Ca^2+^]_o_) indirectly regulates a non-selective cation channel (NSCC) in the vast majority of neocortical nerve terminals via an unknown [Ca^2+^]_o_ sensor [6]. However the mechanism by which [Ca^2+^]_o_ exerts these effects is poorly understood. Recent studies have underlined the central role in regulation of neurotransmission of a number of calcium signaling pathways [1], [7]. While synchronous, asynchronous and spontaneous transmitter release have all been shown to strongly depend on extracellular Ca^2+^ [7], [8], [9] these effects are usually attributed to Ca^2+^ entry via voltage-activated Ca^2+^ channels (VACC) and less attention has been focused on the presynaptic role of other Ca^2+^ signaling pathways, such as surface charge screening [10], [11], [12] or Ca^2+^-dependent ion channels [6], [13], [14]. Attention is returning to these other pathways with the realization that their influence may have been underappreciated because supraphysiological [Ca^2+^]_o_ employed in most studies ensured maximal receptor activation and decreased the impact of physiological decreases in external [Ca^2+^].

In this study we identify the receptor activating a novel calcium signaling pathway using direct patch clamp recordings from nerve terminals. Candidate [Ca^2+^]_o_ receptors include the extracellular calcium-sensing receptor (CaSR), metabotropic glutamate receptor (mGluR) and γ-aminobutyric acid B receptor (GABA_B_R). All of these receptors have been identified as sensitive to [Ca^2+^]_o_ [15], [16], [17], [18], have been localized to the synapses of central neurons [19], [20], [21], [22], [23], and have been classified as members of G-protein coupled receptor (GPCR) family C [24]. In addition, CaSR may heterodimerize with mGluR and GABA_B_R [25], [26], [27], raising the possibility that heterodimers involving some or all of these GPCRs may modulate NSCC currents in nerve terminals.

Our experiments, studying the impact of CaSR agonists and a CaSR mutation on the [Ca^2+^]_o_ sensor-NSCC pathway in nerve terminals, show that the calcium-sensing receptor (CaSR), the mainstay in serum calcium homeostasis, is the extracellular calcium sensor regulating NSCC activity in neocortical nerve terminals. In contrast, nerve terminals were insensitive to glutamate and GABA arguing strongly against the mGluR and the GABA_B_R mediating these effects. This approach provides insight into a novel pathway through which [Ca^2+^]_o_ influences nerve terminal excitability.

## Results

### CaSR Mutation Reduces Affinity of [Ca^2+^]_o_ Detector

**Figure 1:**
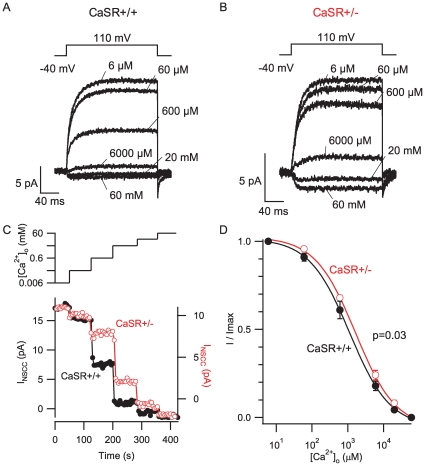
Loss of function CaSR mutation reduces NSCC current sensitivity to [Ca^2+^]_o_. Cell- attached recordings were made from (A) CaSR^+/+^ and (B) CaSR^+/−^ terminals and the Ca^2+^ in the bath solution applied to terminals varied between 6 µM and 60 mM as indicated. Step depolarizations (−40 to 110 mV relative to resting membrane potential) were made every 5 seconds. Average current traces (n = 8−15) are shown for each bath [Ca^2+^] at steady-state for two exemplar recordings. Note that outward currents elicited with 0.6 and 6 mM Ca^2+^ were proportionately larger in the heterozygote than in the wild-type recording. C) timecourse of NSCC current amplitude (measured at the end of the depolarizing step) in the same CaSR^+/+^ (filled circles, left axis) and CaSR^+/−^ (open circles, right axis) terminals as bath [Ca^2+^] was increased (upper trace). Steady state amplitude was reached in 5–10 s for both CaSR^+/+^ and CaSR^+/−^ genotypes. Axes were scaled to span the current amplitudes measured between with bath [Ca^2+^] between 6 µM and 60 mM. D, the concentration-effect relationship for both CaSR genotypes shows that wild-type terminals exhibited higher affinity for Ca^2+^ (p = 0.032). NSCC currents were normalized for each terminal by measuring the difference between the NSCC current and the 60 mM Ca^2+^-elicited NSCC current and dividing this by the difference between the NSCC currents elecited by 6 µM and 60 mM Ca^2+^. The curves represent mean±SEM of 7 and 6 recordings for CaSR^+/+^ and CaSR^+/−^, respectively. The curves were fit to the average data points resulting in Hill coefficients of 0.77 for both genotypes and IC_50_s of 1.6±0.2 mM and 1.1±0.07 mM for CaSR^+/−^ and CaSR^+/+^, respectively.

Small, acutely isolated neocortical nerve terminals sense [Ca^2+^]_o_ and indirectly modulate a NSCC current (Figure 1A) as reported previously [6], [28]. The CaSR^−/−^ mutant mouse lacks CaSR exon 5 which results in a reduced affinity for Ca^2+^ [29]. However, CaSR^−/−^ mutant mice die prematurely and exhibit delayed growth, preventing the preparation of synaptosomes from these animals. We therefore used the heterozygous mouse (CaSR^+/−^), which has an elevated serum [Ca^2+^] reflecting lower affinity for [Ca^2+^]_o_ but normal growth and survival [30], to examine if the CaSR mutation impacts nerve terminal sensitivity to [Ca^2+^]_o_. In cell-attached recordings, 71 of 76 mouse neocortical nerve terminals (93%) possessed the characteristic [Ca^2+^]_o_-modulated outward current. Outward NSCC currents were activated by depolarization from −40 mV to 110 mV (all voltages were relative to the resting membrane potential) while [Ca^2+^]_o_ was changed between 6 µM and 60 mM (Figure 1A,B). Outward currents were larger at lower [Ca^2+^]_o_. The CaSR^+/−^ terminals were less sensitive to increases in [Ca^2+^]_o_ as illustrated by the traces activated with 0.6 and 6 mM Ca^2+^ in the bath solution (Figure 1A–C). The activation kinetics (Figure 1A,B) were similar for CaSR^+/+^ and CaSR^+/−^ terminals. The concentration-effect relationship for the normalized NSCC current amplitudes (Figure 1D) confirmed that CaSR^+/−^ terminals had a lower affinity than the CaSR^+/+^ terminals (IC_50_ 1.6±0.2 mM versus 1.1±0.07 mM respectively; ANOVA, p = 0.032). We measured the NSCC current amplitudes elicited by 6 µM and 60 mM bath Ca^2+^ to test if the maximum and minimum currents respectively were also dependent on genotype. However, CaSR^+/+^ and CaSR^+/−^ terminals had similar maximum (20±5 pA, n = 16 vs 17±4 pA, n = 13; p = 0.61) and minimum currents (−4.5±3.5 pA, n = 7 vs −3.3±2.1 pA; p = 0.79) indicating the IC_50_, but not NSCC current amplitude was dependent on the CaSR genotype. The reduction in affinity for [Ca^2+^]_o_ in CaSR+/− terminals was consistent with CaSR involvement in modulation of the NSCC currents. The relatively modest shift in affinity is similar to the changes observed following heterologous co-expression of normal and other mutant CaSR [31].

### CaSR Is Present in Nerve Terminals

**Figure 2:**
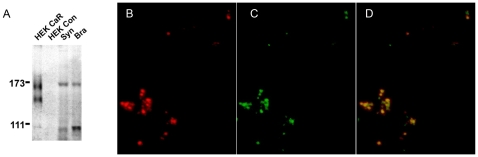
CaSR is present in nerve terminals of neocortex. A, immunoblot of synaptosomes and whole brain show 160 kDa bands with anti-CaSR antibody [76]. Positive control (HEK CaR) shows 140 and 160 kDa bands (glycosylated and unglycosylated forms) in CaSR- transfected HEK cells and no signal in untransfected HEK cells (HEK Con). B, acutely isolated nerve terminals (synaptosomes) identified using the synaptophysin antibody (red). C, CaSR identified with polyclonal antibody “4641” (green). D, superimposition of B and C shows that CaSR and synaptophysin are co-localized.

We next tested that CaSR was present in neocortical nerve terminals using immunochemical techniques. CaSR was present in rat whole brain tissue and synaptosomes by immunoblotting. Western blots detected 140 and 160 kDa bands in HEK cells transfected with CaSR (Figure 2A, left lane). These have been shown to represent differentially glycosylated forms [29] and were absent in untransfected control cells (Figure 2A, second lane). Synaptosomes and whole brain (Figure 2A, third and fourth lanes, respectively) contained the 160 kDa band and a lower band at 90 kDa, both of which were absent in control experiments following preincubation with the specific antigenic peptide fragment (data not shown). The 90 kDa band which has been reported by others was also blocked by peptide fragment and appears to be due to CaSR degradation [32]. To confirm CaSR was present in nerve terminals we used a polyclonal antibody raised against CaSR (4641; Figure 2C) and co-stained synaptosomes with an antibody to synaptophysin (Figure 2B). Both antibodies gave similar punctate patterns of staining that co-localized (Figure 2D) indicating that CaSR is indeed present in neocortical nerve terminals. The polyclonal antibody 4641 also identified the 140 and 160 kDa bands in Western blots consistent with a specific action (data not shown).

### The Nerve Terminal [Ca^2+^]_o_ Sensor Is Modulated by Allosteric CaSR Agonists

**Figure 3:**
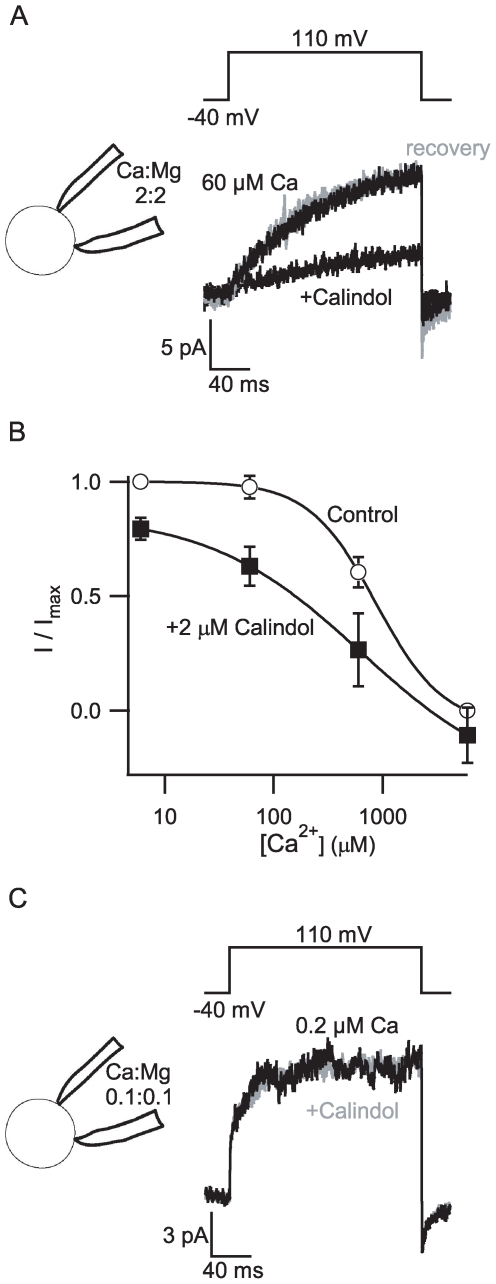
Calindol facilitates inhibition of NSCC currents by extracellular Ca^2+^. A, exemplar traces show NSCC currents reversibly inhibited by addition of Calindol (10 µM) to bath solution. Traces were recorded in cell-attached mode following 200 ms step depolarization with 60 µM Ca^2+^ and 0 Mg^2+^ in the bath and 2 mM Ca^2+^ and 2 mM Mg^2+^ in the pipette solution (inset). The substantial inhibition of NSCC current was reversed following washout (gray trace). B, the Ca^2+^ concentration-effect relationship was left-shifted by the allosteric CaSR agonist Calindol (2 µM). These data represent 11 synaptosome recordings, each normalized to the current observed in 6 µM Ca^2+^. C, average traces show NSCC currents unaffected by the addition of 10 µM Calindol (gray trace) to bath solution containing reduced [Ca^2+^]_o_. Traces were recorded in cell-attached recording following 200 ms step depolarization with 0.2 µM Ca^2+^ and 0 Mg^2+^ in the bath and 0.1 mM Ca^2+^ and 0.1 Mg^2+^ in the pipette solution (inset). Recordings were less stable at low divalent concentrations; traces are thus averages of 8 currents elicited with a 5 second duty cycle.

CaSR agonists including Ca^2+^, Mg^2+^, spermidine, gadolinium, and neomycin have been shown to modulate NSCC currents in nerve terminals [6], [33]. However, since these CaSR agonists also interact with other targets [34], we employed more specific pharmacological interventions against CaSR to explore the identity of [Ca^2+^]_o_-modulated NSCC. Allosteric CaSR agonists bind to a transmembrane pocket of CaSR [35], [36]. We tested if the [Ca^2+^]_o_-modulated NSCC currents in rat synaptosomes were sensitive to Calindol, an allosteric CaSR agonist [37]. Out of 290 cell-attached recordings from rat neocortical nerve terminals, 230 (79%) possessed the characteristic [Ca^2+^]_o_-modulated outward current. In the cell-attached configuration, NSCC currents activated by depolarization with a [Ca^2+^]_o_ of 60 µM were substantially and reversibly inhibited by bath application of 10 µM Calindol (Figure 3A). Control experiments showed that the solvent (ethanol) had no effect on the NSCC current amplitude at 0.1% (Figure S1). Calindol acted in an apparently allosteric fashion shifting the concentration-effect relationship for [Ca^2+^]_o_ to the left. The average data from 11 recordings (Figure 3B) revealed a decrease in slope as well as a left-shift with 2 µM Calindol. To test whether the effect of Calindol was reduced in the presence of very low [Ca^2+^] as expected for an allosteric agonist, we reduced the bath [Ca^2+^] to nominally 0.2 µM and patch pipette solution [Ca^2+^] and [Mg^2+^] from 2 mM to 100 µM. Under these conditions, Calindol did not affect the NSCC currents (Figure 3C; control versus test: 4.5±2.4 pA versus 4.5±2.6 pA control versus; p>0.05, n = 4). These findings indicate that Calindol modulation of the [Ca^2+^]_o_-modulated NSCC signaling pathway in nerve terminals is allosteric, similar to its action on CaSR in heterologous expression systems [38].

### Calindol Activation of Terminal CaSR Exhibits Substantial Delay

**Figure 4:**
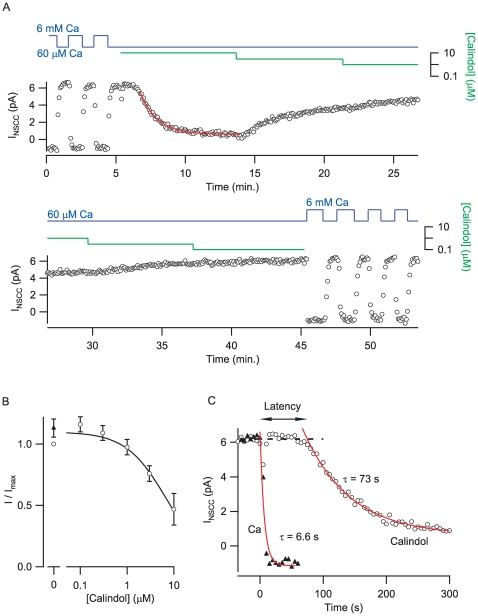
Kinetics and dose-dependence of Calindol inhibtion of NSCC currents in synaptosomes. A, contiguous plot of NSCC current amplitude versus time showing timecourse of on and off kinetics for Calindol action in a cell-attached synaptosome recording. The outward current elicited by voltage step (200 ms, −40 to 110 mV) every 5 seconds is plotted against time. NSCC current was reversibly and reliably activated by reductions in bath [Ca^2+^] (0.06–6 mM; blue trace) and inhibited by Calindol (0.1–10 µM; green trace). The Ca^2+^ and Calindol axes are logarithmic and the absence of the green trace indicates Calindol concentration is zero. Calindol (10 µM) inhibited NSCC current after a 1–2 minute delay and thereafter blocked with mono-exponential time course. The current increased with an exponential time course following reduction of Calindol. B, Calindol inhibited NSCC currents in bath [Ca^2+^] of 60 µM with an IC_50_ 6.3±1.1 µM on average (n = 4). Each recording was normalized by dividing the NSCC current amplitude by the NSCC current amplitude elicited by 60 µM bath [Ca^2+^] before Calindol was applied. Calindol was generally applied at higher concentrations first and the amplitude of the NSCC current increased after washout (closed triangle) compared to initial baseline (open circle). This presumably reflects a run-up phenomenon due to the long duration of these experiments. C, Calindol inhibition of NSCC current is biphasic (i.e. latency and monoexponential) whereas Ca^2+^ inhibition is well described by a single exponential. Two sections of the timecourse data in A displaying applications of 10 µM Calindol and 6 mM Ca^2+^ (just prior to Calindol application) were redrawn on expanded time axis and overlaid so that time zero corresponded to solution change. Both datasets are well-fit by single exponentials with Calindol decaying significantly more slowly (tau of 73 s for Calindol vs 6.6 s for Ca^2+^) and at a substantial latency.

We next examined the potency and kinetics of action of Calindol on the NSCC currents in synaptosomes. Cell-attached recordings were made from synaptosomes and the membrane depolarized from −40 to 110 mV every 5 seconds. In the exemplar recording, the current activated by a 200 ms depolarization briskly and reliably increased to ∼6 pA following a decrease in bath [Ca^2+^] (blue line) from 6 mM to 60 µM (Figure 4A). Addition of Calindol (10 µM), denoted by the green line, slowly decreased the NSCC current amplitude seen with 60 µM Ca^2+^ in the bath. Subsequent decreases in Calindol (green line; range 0.1 to 10 µM) resulted in increases in NSCC current amplitude at a fixed bath [Ca^2+^] (blue line). Like the initial decrease in response to Calindol, the other changes in NSCC current amplitude were well described by exponential time courses. The average IC_50_ for Calindol was 6.3±1.1 µM at 60 µM bath [Ca^2+^] (n = 4, Figure 4B). The NSCC current amplitude tended to “run-up” during these prolonged recordings and consequently the NSCC current amplitudes for the wash (solid triangle) and the lower doses of Calindol were slightly larger than the control currents (Figure 4B).

Closer inspection of the NSCC current-time plots (Figure 4C) shows the kinetics for the Calindol-induced and Ca^2+^-induced reductions in current amplitude were different. The expanded and superimposed time scales illustrate that the response to Ca^2+^ was much faster than the response to Calindol (Figure 4C). Moreover, inhibition of the NSCC current by Calindol occurred after a substantial delay (75 sec) which sharply contrasted with rapid onset of inhibition of NSCC currents when Ca^2+^ was increased from 60 µM to 6 mM.

**Figure 5:**
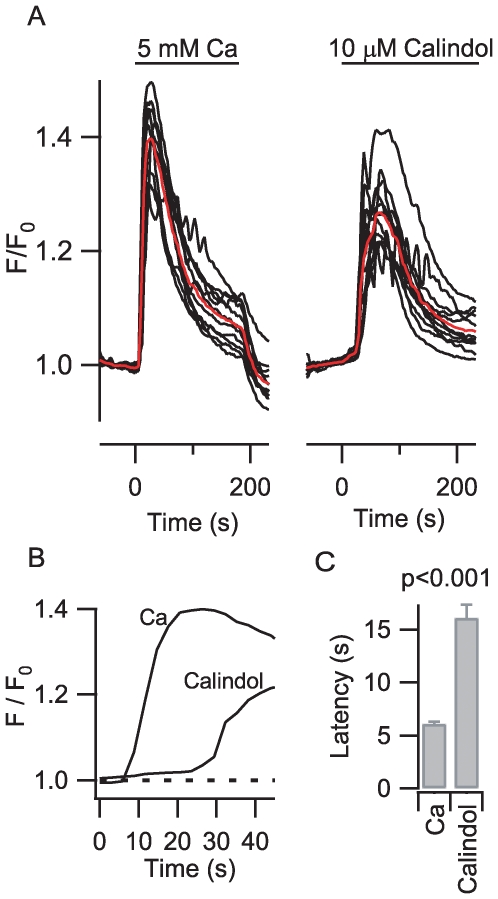
Calindol activation of CaSR expressed in HEK cells occurred with a greater latency than Ca^2+^ activation of CaSR. A, application of Ca^2+^ (5 mM) and Calindol (10 µM) at time zero to CaSR-expressing HEK cells in 1 mM Ca^2+^ and 0 Mg^2+^ caused a transient increase in fluorescence (F) relative to basal level (F_0_) indicating an increase in [Ca^2+^]_i_. The black curves denote signal from 12 cells and the red curves indicate the average. There was a 5 minute delay between applications. B, average curves from A have been redrawn on the same time-expanded axis to compare effect latency of Calindol vs. Ca^2+^. C, histogram of latency of effect for Ca^2+^ and Calindol. The latency was significantly greater for activation by Calindol (23.7±1.2 s) than by Ca^2+^ alone (9.0±0.2 s) in the recordings from 22 cells (p<0.001).

Calindol has not previously been reported to exhibit any delay or latency of action. If Calindol's action on the [Ca^2+^]_o_-modulated NSCC signaling pathway in small nerve terminals was mediated via CaSR we reasoned that this latency should also be present in measurements using heterologously expressed CaSR. We tested this idea by studying CaSR modulation in transiently transfected HEK cells. In this expression system stimulation of CaSR activates phospholipase C, increasing inositol triphosphate production and release of Ca^2+^ from intracellular stores [39]. Transfected cells were identified via fluorescence of cotransfected EGFP. Changes in [Ca^2+^]_i_ were measured via the increase in X-rhod1 fluorescence above baseline (F/F_0_). Increasing bath [Ca^2+^] from 1 to 5 mM (at t = 0) resulted in an average increase in F/F_0_ of 0.400±0.017 (n = 12; individual and average responses represented by black and red traces respectively). After a five minute wash in 1 mM Ca, application of Calindol (10 µM) resulted in a F/F_0_ rise of 0.269±0.017 in the same 12 cells (Figure 5A). Superimposing the time courses of effects shows that the response to Calindol occurs after a substantially larger lag (Figure 5B) than that observed after activation by Ca^2+^. Measurement of the latency (by deflection >4 S.D. above baseline noise) showed a substantial increase with Calindol compared to 5 mM Ca^2+^ (9.0±0.2 s vs 23.7±1.2 s, n = 22; p<0.001) in heterologously expressed CaSR consistent with our observation at isolated small terminals.

Previously, NSCC currents in isolated nerve terminals have been reported to be insensitive to the weak CaSR agonist NPS-467 [6]. In light of the latency of action of Calindol (Figure 4C) we re-tested whether NPS-467 was effective on nerve terminals using longer applications and higher agonist concentrations. Application of NPS-467 (10 µM) inhibited 34±5% (n = 4) of the NSCC current activated by 60 µM Ca^2+^ in cell-attached recordings from synaptosomes (Figure S2). The high NPS-467 concentration required is consistent with other reports using expressed CaSR [40].

**Figure 6:**
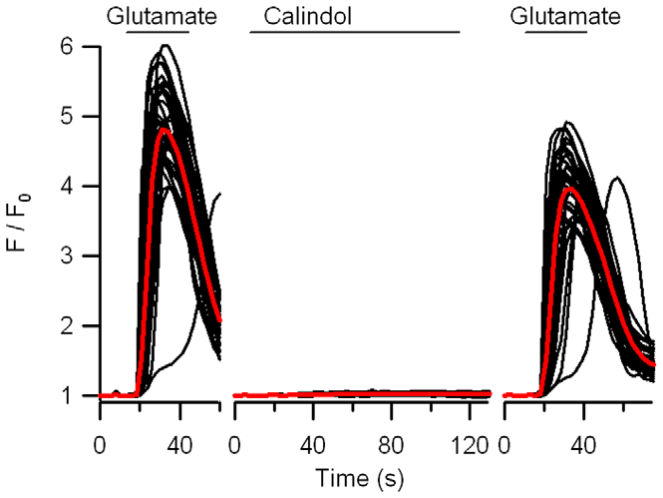
Glutamate (10 µM) but not Calindol (5 µM) activated the mGluR1 expressed in HEK cells. Application of glutamate (horizontal bars) caused a transient increase in fluorescence (F) relative to basal level (F_0_) indicating an increase in [Ca^2+^]_i_ (left). The black curves denote signals from 35 cells while red curves indicate averages. Applications of test solutions were staggered by 10 min to allow for recovery. Calindol application (horizontal bar) to the same cells did not produce any change in F/F_0_ (middle), yet these cells remained responsive to glutamate (right).

CaSR and mGluR1 are both family C GPCRs and although they only have an amino acid identity <24% they are similar in terms of their large extracellular domains, tendency to function as dimers, and because they both possess a membranous binding pocket [35], [41]. Since Calindol binds to a membranous pocket [36], we asked whether the effects of Calindol on synaptosomes were mediated via mGluR. Using mGluR1-expressing HEK cells (kind gift of Dr J. Saugstad) we tested if Calindol also activated mGluR. Bath application of 10 µM glutamate increased F/F_0_ on average by ∼4 (red trace is average of 30 cells each represented by black trace) in the mGluR1 cells (Figure 6). However, the same cells did not respond to Calindol (5 µM) when applied 10 minutes later in the presence of 1 mM [Ca^2+^]_o_. The absence of effect was not due to receptor desensitization or depletion of Ca^2+^ from intracellular stores as glutamate evoked a similar response when reapplied after an additional 10 minutes. Untransfected HEK cells did not respond to glutamate or Calindol (data not shown).

These data strongly support the proposal that the [Ca^2+^]_o_-modulated NSCC signaling pathway in nerve terminals involves CaSR. In addition, the distinct kinetics of action for Calindol and Ca^2+^ confirm different mechanisms of action for these two classes of CaSR agonist.

### Nerve Terminal [Ca^2+^]_o_ Detector Not Modulated by Glutamate or GABA

**Figure 7:**
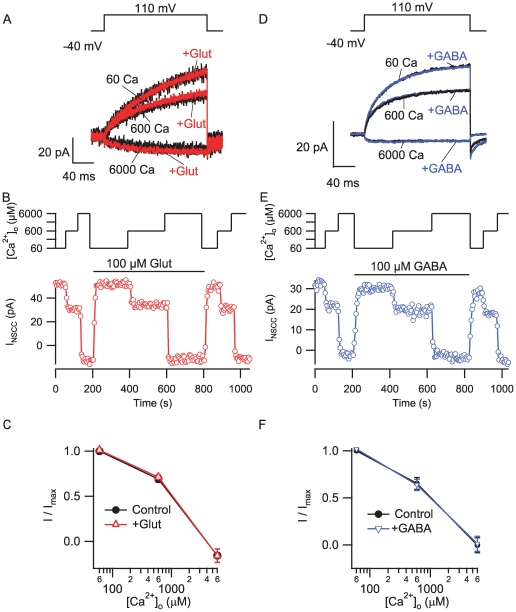
The [Ca^2+^]_o_-modulated NSCC current in rat nerve terminals was unaffected by glutamate and GABA. A, currents activated by step depolarizations (−40 to 110 mV relative to membrane potential) across a range of bath [Ca^2+^] (60 µM-6 mM, black traces) superimposed with those recorded during addition of 100 µM glutamate to the perfusate (red traces). Each trace represents an average of 10–20 currents at steady state solution conditions from the same cell-attached recording from a synaptosome. B, timecourse of NSCC current amplitude activated by the 200 ms voltage step every 5 seconds at three [Ca^2+^]_o_ (upper trace). Same recording as A. Glutamate application is denoted by horizontal bar. C, normalized concentration-effect relationship for changes in [Ca^2+^]_o_ from NSCC synaptosome recordings is unaffected by glutamate (n = 6). Currents represent NSCC current amplitude at end of voltage step relative to current before step divided by NSCC current amplitude with 60 µM Ca^2+^ in bath. D, currents activated by step depolarizations over a range of bath [Ca^2+^] (60 µM-6 mM, black traces) superimposed with those recorded during perfusion of 100 µM GABA (red traces). Each trace represents an average of 10–20 currents at steady state solution conditions from the same cell-attached recording from a synaptosome. E, timecourse of NSCC current amplitude from D at three [Ca^2+^]_o_ (upper trace). GABA application is denoted by horizontal bar. E, normalized concentration-effect relationship for [Ca^2+^]_ o_ and NSCC amplitude in synaptosome recordings is unaffected by GABA (n = 3). Currents represent NSCC current amplitude at end of voltage step relative to current before step divided by NSCC current amplitude with 60 µM Ca^2+^ in bath.

Initially, CaSR, mGluR and GABA_B_R were all possible candidates for the neocortical [Ca^2+^]_o_ sensor as they are sensitive to [Ca^2+^]_o_ [15], [16], [17], [18] and localized to the synapses of central neurons [19], [20], [21], [22], [23]. Consequently, we addressed if mGluR or GABA_B_R were mediating part of the response of the terminals to changes in [Ca^2+^]_o_. We addressed this issue by testing whether glutamate or GABA modulate the NSCC currents in isolated rat neocortical nerve terminals. In cell-attached recordings, depolarization from −40 to 110 mV activated outward currents through NSCCs that increased as bath [Ca^2+^] was reduced (Figure 7). Co-application of 100 µM glutamate did not affect the amplitude or rate of activation of the NSCC currents (Figure 7A, red trace). In addition, glutamate did not appreciably slow the response of the terminal to changes in [Ca^2+^]_o_ which is illustrated in the plot of NSCC current amplitude versus time (Figure 7B). The absence of an effect of glutamate was confirmed in recordings from 11 terminals (Figure 7C).

Brain extracellular glutamate concentration varies between 25 nM at rest [42] and 1–5 mM following exocytosis [43]. While these higher levels are short-lived and thus less likely to affect GPCR signaling, we tested whether they could impact the NSCC current in nerve terminals. Glutamate (2 mM) was ineffective at altering the size or kinetics of the Ca-modulated currents in rat terminals (n = 5; Figure S3).

We also tested the action of GABA (100 µM) in a parallel set of experiments (n = 3). GABA was equally ineffective at altering current amplitude, activation or responsiveness to [Ca^2+^]_o_ (Figure 7D–F). The absence of any effect of glutamate or GABA effectively rules out the mGluR and GABA_B_ receptor as candidate [Ca^2+^]_o_ sensors that modulate NSCC currents in small neocortical terminals.

## Discussion

Sustained decreases in [Ca^2+^]_o_ will depress subsequent synaptic transmission due to the exquisite sensitivity of transmitter release to [Ca^2+^]_o_ [8], [44] following its entry via VACC [45], [46]. A pathway that detects such falls in [Ca^2+^]_o_ and indirectly activates a voltage-dependent NSCC was identified in nerve terminals in the neocortex [6]. Here we described experiments that identify CaSR as the [Ca^2+^]_o_-sensor in the [Ca^2+^]_o_-sensor-NSCC signaling pathway in neocortical terminals. Consequently presynaptic CaSR is positioned to alter presynaptic excitability and attenuate the impact of falls in [Ca^2+^]_o_ on synaptic transmission [28]. In addition, our experiments led us to two further unexpected conclusions. First, direct and allosteric CaSR agonists have distinct kinetics of action. Second, glutamate and GABA do not modulate the sensitivity of the nerve terminals to changes in [Ca^2+^]_o_ suggesting the previously isolated CaSR-mGluR and CaSR-GABA_B_R heterodimers may operate in alternative subcellular compartments or in other brain regions.

### CaSR Is the Nerve Terminal [Ca^2+^]_o_ Sensor

We demonstrated that CaSR is present in nerve terminals by immunoblot and immunofluorescence, that [Ca^2+^]_o_ has reduced potency at terminals from heterozygous mutant CaSR- containing mice, and that Calindol modulates terminal NSCC currents. These data strongly support the hypothesis that the mechanism by which changes in [Ca^2+^]_o_ are transduced in neocortical nerve terminals involves CaSR. Moreover, the lack of any action of glutamate or GABA on the NSCC current indicate that neither mGluR nor GABA_B_R act as neocortical nerve terminal [Ca^2+^]_o_-sensors that regulate the NSCC current.

The [Ca^2+^]_o_-sensor-NSCC signaling pathway was present in the vast majority of neocortical nerve terminals from mouse (93%) and rat (79%) consistent with a pervasive role in neuronal signaling. The IC_50_ of Ca^2+^ inhibition of mouse neocortical terminal NSCC current (1.1 mM) was lower than the EC_50_ for Ca^2+^ activation of heterologously expressed CaSR (1.75–4.1 mM) [31], [39], [47], [48]. There are a number of possible explanations for this apparent difference. First, both sets of measurements detected different functional changes downstream of CaSR and thus any non-linearities in the signaling pathways would change the apparent affinity for Ca^2+^. Second, desensitization has also been shown to alter the CaSR concentration-effect relationship and reduce the apparent affinity of Ca^2+^ [39]. Regulation of CaSR desensitization is not fully understood [39], [49]. However, in our experiments in nerve terminals, evidence of desensitization was only present in experiments lasting >30 minutes (Figure 4); NSCC current amplitudes were generally stable over periods shorter than this (Figure 7B,E). This contrasted with the relatively transient nature of the rise of [Ca^2+^]_i_ in CaSR expressing HEK cells (Figure 5) and may in part explain differences in apparent affinities between preparations. Third, the discrepancies in affinity may arise from differences between expression systems and native tissue receptors resulting from post-translational modification or protein-protein interactions. Consistent with this an increased affinity has been reported for CaSR signaling in glial cells [50]. The IC_50_ of [Ca^2+^]_o_ inhibition of terminal NSCC current shifted from 1.1 to 1.6 mM (Figure 1; CaSR^+/+^ and CaSR^+/−^ respectively). This modest shift in affinity is in line with the changes reported following heterologous co-expression of normal and mutant CaSR [31] and may be attributed to partial rescue of CaSR mutants by wild-type receptor (Wang and Smith, unpublished observations). The small difference in affinity is reflects the mildness of the phenotype of the heterozygote mouse; the serum ionized calcium levels in CaSR^+/+^ and CaSR^+/−^ mice are 1.2±0.1 and 1.4±0.0 mM, respectively [30].

### Latency of Calindol Action

Calindol is an allosteric agonist that binds to a relatively inaccessible membranous pocket on CaSR, increasing the sensitivity of the receptor to Ca^2+^ [36]. The striking difference between both agonists in the time to onset of action (Figs. 4C,5B) presumably reflects differences in the mechanism of action of Ca^2+^ and Calindol. The much longer latency for Calindol may reflect the time taken for it to reach and bind to the less accessible membranous pocket and/or the time taken for CaSR to alter conformation once Calindol is bound. Inhibition of NSCC currents by Calindol exhibited a slower exponential rate compared to when Ca^2+^ was applied at 6 mM (Figure 4C). As the bath [Ca^2+^] was 60 µM during the Calindol application, the ten-fold decrease in the rate of NSCC current block may be postulated to reflect the reduced rate of Ca^2+^ binding to CaSR. However this explanation is unsatisfactory as Ca^2+^-mediated NSCC current block was more rapid than Calindol-mediated block even when much lower [Ca^2+^]_o_ was employed (Figures 2C & 7B,E). An alternative explanation is that the second phase (exponential decline) of NSCC current block by Calindol may in part be attributed to conformational changes. Further experiments are required to test these hypotheses. The latency for Calindol was smaller in CaSR-transfected HEK cells than in synaptosomes and may reflect differences in the signaling downstream of CaSR.

### Heterodimers

CaSR exists as homodimers which form in the endoplasmic reticulum before transportation to the cell membrane [51]. Heterodimers comprised of CaSR and other GPCRs have also been identified [27], [52]. Howevere these data make it unlikely that Ca^2+^-modulated currents in rat nerve terminals are mediated by heterodimers composed of CaSR and mGluR or GABA_B_R as CaSR-mGluR heterodimers retain sensitivity to glutamate [27]. Our data do not exclude the possibility that CaSR is forming heterodimers with other GPCRs. GPRC6A, a recently identified group C GPCR, is one potential candidate that may heterodimerize with CaSR based on similarities in structure and sensitivity to [Ca^2+^]_o_ [53], [54].

### Function of Nerve Terminal CaSR

CaSR is associated with epilepsy [55] and dementia [56] however the consequences of CaSR activation in the CNS have not been fully characterized. In contrast, CaSR has been intensively studied in the periphery due to its central role in systemic calcium homeostasis [57]. CaSR activation by external Ca^2+^ regulates parathyroid hormone and calcitonin secretion from the parathyroid and thyroid glands, respectively, and thus maintains serum [Ca^2+^] [58], [59], [60]. Brain extracellular [Ca^2+^] is dynamic and decreases as neuronal activity increases- moderate decreases in [Ca^2+^]_o_ accompany bursts of synaptic activity [61] while profound falls to ∼0.1 mM result from focal brain trauma or ischemia [62], [63]. These falls in [Ca^2+^]_o_ will reduce stimulation of CaSR and thereby increase NSCC activity in the vast majority of neocortical nerve terminals [28]. Our current model proposes that decreases in cleft [Ca^2+^] will reduce Ca^2+^ entry and synaptic transmission [8] but that the fall in cleft [Ca^2+^] will act as feedback to presynaptic CaSR. Increased NSCC activity may increase action potential duration [64], prolong Ca^2+^ entry at the terminal and thus increase release probability [65]. Consequently CaSR may be operating as part of a homeostatic pathway to prevent synaptic failure when [Ca^2+^]_o_ falls [28].

Alterations of such a homeostatic pathway could lead to increased excitability and gain-of-function CaSR mutations have been associated with childhood epilepsy [66]. One possible explanation is that the increased calcium affinity of gain-of-function CaSR mutants may steepen the response to normal changes in [Ca^2+^]_o_, lead to overcompensation of the homeostatic pathway and increase the probability of aberrant activity. Consistent with this, loss-of-function CaSR−/− mutant neurons have a more negative resting membrane potential [28] which could reduce the likelihood of seizures. However, the CaSR−/− mutation increases release probability at excitatory synapses at basal [Ca^2+^]_o_ [28] which is expected to increase neuronal excitability and increase the risk of epilepsy. Clearly additional study is required to determine how CaSR mutations lead to epilepsy [55].

If it is so widespread in the neocortex why has the impact of CaSR signaling been overlooked? Previous studies examining external Ca^2+^ on synaptic transmission may have attenuated the impact of the CaSR-NSCC pathway by using non-physiological high resting [Ca^2+^]_o_ and by increasing bath [Mg^2+^] as bath [Ca^2+^] was decreased [8], [67], [68]. The use of physiological [Ca^2+^]_o_ has unmasked other, previously overlooked signaling pathways that modulate synaptic transmission [69]. In fact, both recurrent rhythmic activity and repetitive synaptic transmission in brain slices more closely resembled findings *in vivo* when slice [Ca^2+^]_o_ was reduced from the commonly used 2 mM to the more physiological 1.0–1.2 mM [70], [71]. It remains to be determined how much of the change in neuronal function associated with use of physiological [Ca^2+^]_o_ arises from a reduction in CaSR activation.

GPCR promiscuity [72], [73], the large number of downstream CaSR signaling pathways [74], and its presence in non-neuronal brain tissue [50], [75] all point to CaSR potentially having additional actions in the brain. Further experiments are required to elucidate these other actions.

### Conclusions

Decreases in [Ca^2+^]_o_ will depress synaptic transmission because of the exquisite sensitivity of transmitter release to [Ca^2+^]_o_ [8], [44]. Our experiments identify CaSR as a key component in the [Ca^2+^]_o_-sensor-NSCC signaling pathway in neocortical terminals that may alter terminal excitability. The prevalence of CaSR in neocortex indicates that this signaling pathway may have wide-ranging influence on synaptic transmission during normal function and disease states.

## Materials and Methods

### Synaptosome

All animal procedures were approved by OHSU I.A.C.U.C. in accordance with the U.S. Public Health Service Policy on Humane Care and Use of Laboratory Animals and the N.I.H. Guide for the Care and Use of Laboratory Animals. Male or female Sprague-Dawley rats or 129S6/SvEv mice (six to eight weeks old) were deeply anesthetized with isoflurane and decapitated. As described previously [28], the neocortex was submerged in ice-cold 320 mM sucrose solution in a Teflon-glass tissue homogenizer driven at 400–500 RPM, then centrifuged at 3000 g for 3 minutes. The supernatant was centrifuged at 14600 g for 12 minutes and the upper layer of the resulting pellet resuspended in approximately 2 ml ice-cold sucrose. Before use the synaptosomes were washed with Tyrode solution.

### Genotyping CaSR Mutant Mice

DNA from mouse tail samples was released by treatment with 50 mM NaOH at 95°C for 15 minutes, followed by the addition of 1 M Tris buffer, pH 8.0, containing 10 mM EDTA. Polymerase chain reaction was then performed using DNA solution and three primers: CaSR 5′: TCTCTTCTCTTTAGGTCCTGAAAGA, CaSR 3′: TCATTGATGAACAGTCTTTCTCCCT, and r-neo2: TCTTGATTCCCACTTTG TCCT TG TA. The samples were run on a 1% agarose gel and the sample identified as CaSR^+/+^, CaSR^+/−^, or CaSR^−/−^.

### HEK Cell Culture and Transient Transfection

HEK 293 cells were maintained in Dulbecco's modified Eagle's medium supplemented with 5% fetal bovine serum (FBS). For intracellular [Ca^2+^] ([Ca^2+^]_i_) imaging experiments, ∼10^5^ HEK cells were plated in 500 µl medium onto poly-D-lysine-coated glass coverslips in 24-well plate. Cells were transfected with pEGFP/CaSR plasmid DNA using Lipofectamine one day after plating. Briefly, 0.8 µg pEGFP/CaSR plasmid DNA was mixed in 50 µl Opti-MEM® I Reduced Serum Medium and 10 µl Lipofectamine 2000 (Invitrogen) mixed in 50 µl Opti-MEM® I Reduced Serum Medium. The mixtures were incubated for 20 minutes and then added to each well containing cells and medium. The transfection medium was replaced with DMEM containing 5% FBS, six hours later. Cells were kept in culture for another 18 to 24 hours before use. Untransfected cells were used in control experiments 42–48 hours after plating. Stable lines of HEK cells containing mGluR1 were used once confluent.

### Immunofluorescence

Synaptosomes were fixed for 30 minutes at 4°C with 4% formaldehyde, washed with PBS, and then placed in blocking solution containing 2% goat serum, 1% BSA and 0.4% saponin for 30 mins at 25°C. Overnight incubation in primary antibody-containing solution (4641, polyclonal antibody kindly provided by Dr E. Nemeth of NPS Pharmaceuticals [31] or a monoclonal antibody against synaptophysin, MAB5258 Chemicon) at 4°C was followed by a wash and then 30 minute incubation with secondary antibodies at 37°C. Coverslips were then washed and mounted, together with a quenching agent (Citifluor), and viewed by fluorescence microscopy.

### Western Blots

Brains were harvested, flash frozen, and homogenized in a protease inhibitor-containing lysis buffer (10 µg/mL aprotinin, 10 µg/mL pepstatin A, 10 µg/mL leupeptin, 10 µg/mL benzamidine, and 1 mM PMSF) on ice. Protein concentration was determined by the Bradford technique. Membrane fragments were run on a 7% polyacrylamide gel and then transferred overnight. Blots were blocked with 3% albumin and then stained with a polyclonal anti-CaSR antibody [76] at 1 in 1000 dilution (kindly provided by Dr K Rodland) and with secondary antibodies conjugated with horseradish peroxidase at 1 in 50,000. In control experiments the primary antibody was preincubated with 50 µg/mL blocking peptide. Bands were visualized with chemiluminescence.

### Electrophysiology

Recordings were made from acutely isolated single nerve terminals (synaptosomes) visualized using an inverted microscope (IX70, Olympus) as previously described [6]. Electrodes with resistances of 15–40 MΩ were used to make cell-attached recordings from terminals. Test solutions were continuously applied from a nearby capillary tube and manifold at 1–3 ml/min (23–25°C). We employed a Tyrode solution (in mM; 150 NaCl, 4 KCl, 2 CaCl_2_, 2 MgCl_2_ 10 HEPES, and 10 glucose, at pH 7.35 with NaOH) in the bath and electrode unless otherwise stated. In the mouse synaptosome experiments, when [Ca^2+^] was greater than 0.6 mM, NaCl was decreased isotonically for CaCl_2_. Voltage-clamp recordings were obtained using Pulse software and an EPC- amplifier 9 (Heka Instruments Inc, MA). Leak currents were subtracted with a p/-4 protocol. Currents were prefiltered with a 2 or 5 kHz Bessel filter and digitized at 20–100 µs per point. Calindol stock solution was made up at 10 mM in ethanol and stored as aliquots at −20°C.

### Measurement of [Ca^2+^]_i_ Response

[Ca^2+^]_i_ was measured in HEK cells transfected with CaSR-EGFP or mGluR1 using the fluorescent indicators, X-rhod-1 or Fluo-4 respectively. Briefly, cells were loaded for 30 min at 37°C with 2 µM AM X-rhod-1 or 5 µM AM Fluo-4 in Tyrode solution, and then placed on the stage of an inverted microscope (IX-70, Olympus). Images were acquired using a 1.2 N.A. 63x water immersion or PlanApo 1.42 N.A. 60x oil-immersion objective and cooled CCD camera (Orca-ER, Hamamatsu) with computer controlled shutter (UNIBLITZ VMM-D1, Vincent Associates, Rochester, NY). Fluorophores were excited with a halogen lamp. Fluo-4 was excited at 470–490 nm and fluorescence emission measured via a LP filter 510 nm while X-rhod-1 was excited at 542–582 nm and emission measured at 604–644 nm (TXRED-4040B-OMF-ZERO, Semrock, NY). Images were captured and processed using Wasabi image software. The changes in [Ca^2+^]_i_ are reported as fluorescence ratios (F/F_0_) where F_0_ represents the baseline fluorescence. About 10–20 cells with similar cell surface GFP fluorescence from a single field were analyzed simultaneously using Wasabi software.

### Data Analysis

Data were analyzed using custom macros written in Igor Pro (Wavemetrics, Lake Oswego, OR). As previously dose-response data were fit with the Hill equation where I represented current at concentration A of agonist and I_max_ was the maximum current and n represented the Hill coefficient [77]. NSCC current amplitudes were measured over the last 2–5 ms of a 200 ms depolarizing step. In synaptosome recordings, latency or delay of action of Calindol was measured from the plot of NSCC current amplitude versus time (diary plot) using the interception of the line representing average NSCC current amplitude at 60 µM Ca^2+^ and the extrapolated exponential fitted to the NSCC current during inhibition by Calindol (see Figure 4C). The delay equaled the difference between the intercept and the time at which solutions were switched (t = 0). In experiments using HEK cells the responses were not described by single exponentials and so a different approach was taken. The standard deviation of F_0_ was measured and latency was defined as the time between solution change and the point at which F/F_0_ first deviated above F_0_ by more than four standard deviations.

Tests for paired or unpaired replicates were used as appropriate. Mean±S.E.M. values are reported in general. In the case of nonparametric data, the Mann-Whitney test was used. All tests were two-tailed and a p value<0.05 was considered significant. Graphpad Prism software was used to calculate p-values.

## Supporting Information

Figure S1 NSCC current amplitudes were unaffected by ethanol. Plot of average normalized NSCC amplitude evoked by depolarizing steps (−40 to 110 mV for 200 ms) in the presence of 60 µM bath Ca^2+^ from four recordings. Application of ethanol at 0.1% (black horizontal bar) was associated with a small (<10%), transient (<20 ms) decrease in NSCC current amplitude. At 200 seconds there was no detectable change in the NSCC.
(0.12 MB EPS)

Figure S2 NPS-467 reversibly inhibits NSCC currents in nerve terminal recordings. NSCC currents activated by step depolarizations (−40 to 110 mV relative to membrane potential) in the presence of 60 µM [Ca^2+^]_o_ were decreased by co-application of 10 µM NPS-467. Each trace represents an average of 10–20 currents at steady state solution conditions from the same synaptosome-attached recording. Timecourse of NSCC current amplitude from the same recording at three [Ca^2+^]_o_ (upper trace) revealed that NPS reversibly inhibited the NSCC current amplitude.
(0.28 MB EPS)

Figure S3 The [Ca^2+^]_o_-modulated NSCC current in rat nerve terminals was unaffected by 2 mM glutamate. NSCC currents activated by step depolarizations (−40 to 110 mV relative to membrane potential) over a range of bath [Ca^2+^]_o_ (60 µM-6 mM, black traces) superimposed with those recorded in the presence of 2 mM glutamate (red traces). Each trace represents average of 10–20 currents at steady state solution conditions from the same synaptosome-attached recording.
(0.44 MB EPS)
